# P20 Thoracic outlet obstruction – first presentation of a rheumatological disorder

**DOI:** 10.1093/rap/rkad070.041

**Published:** 2023-09-27

**Authors:** Innocent Munzeiwa, Ira Pande, Asif Ahmad

**Affiliations:** Nottingham University Hospitals, Nottingham, United Kingdom; Nottingham University Hospitals, Nottingham, United Kingdom; Nottingham University Hospitals, Nottingham, United Kingdom

## Abstract

**Introduction:**

Thoracic outlet syndrome presenting in a rheumatology clinic will challenge even the most experienced clinician. We share our experience with a patient presenting initially to the thoracic surgeons with classical symptoms of thoracic outlet obstruction. Workup (imaging) identified a bony swelling at the sternoclavicular region leading to referral to our clinic. Our working diagnosis of this being an extremely rare sole manifestation of a common rheumatological disorder was confirmed. We share our story.

**Case description:**

Mrs LB, a 53 year old female was referred by her GP with a progressive swelling around the right sternoclavicular joint. On workup, we identified that she first presented to the vascular surgeons in 2008 with pain and congestion in her right arm and numbness in the fingers. MR venogram confirmed occlusion of the right subclavian with manoeuvres. She was discharged and managed conservatively with analgesia. This swelling was new, had slowly enlarged over the years and was causing pain in the anterior chest wall. On further questioning she confirmed having pustular psoriasis which was quiescent for 10 years. She denied any other articular (peripheral and spinal) or extra articular symptoms of psoriasis. She had no additional past history.

Examination confirmed a hard 6 cm mass around the sternoclavicular region. The overlying skin was intact. The mass was tender only on movement of the shoulder. The rest of the examination with special focus on skin, neurovascular bundle and system review was unremarkable.

Routine blood tests, inflammatory markers and detailed immunology were unremarkable other than slightly raised IgA 4.3 (range 0.7 – 4.0). X-ray clavicle and localised Computerized tomography (CT) confirmed features in keeping with hyperostosis and periostitis. Magnetic resonance imaging of the whole body identified additional involvement of the thoracic spine with endplate and vertebral body oedema at T3/4, T5/6, T6/7, T8/9 and T9/T10.

Our working diagnosis of this being a rare manifestation of SAPHO was confirmed. She fulfilled the modified diagnostic criteria based on the clinical manifestations of previous pustular psoriasis and osteo-articular features on imaging.

As she had been on non-steroidal anti-inflammatories for the pain around the shoulder with little effect and our workup identified additional extensive subclinical spinal involvement the patient has been started on anti-TNF therapy (adalimumab) with planned interval MRI to monitor disease and assess response.

**Discussion:**

SAPHO syndrome is a rare disorder, which consists of synovitis, acne, pustulosis, hyperostosis and osteitis. As in our case, it is predominant in middle-age women. Immunological, genetic and infectious factors are underlying etiology, but the exact pathophysiological mechanisms is unknown. The innate immune system is likely to be involved via elevation of pro-inflammatory cytokines, including IL-8, IL-17 and TNF-α. The bacterial species Cutibacterium (formerly termed Propionibacterium acnes) has been identified in previous studies. Genetic factors are proposed to be involved but no specific genes have been identified. SAPHO syndrome has varied presentations. All the features of SAPHO may not be present at the same time. A high index of suspicion, careful history, radiological and skin manifestations are key to early diagnosis. As in this case, skin manifestations can predate skeletal manifestations by years. Sternoclavicular joint involvement is common (65-90%) followed by spine (32 – 52%) and pelvis (13 – 52%). On careful review of the chest x-ray from 2008 we concluded that her first presentation with thoracic outlet symptoms was due to SAPHO syndrome. Soft tissue swelling around the area of the clavicle can cause compression of structures before significant changes are seen on X Rays. MRI is recommended as this is more sensitive to pick up asymptomatic and early lesions. Even though this patient did not have any inflammatory spinal symptoms imaging identified multiple sites of active spinal disease. Until now there are no treatment guidelines for SAPHO syndrome. It is generally recommended that where NSAIDS have failed and there is axial involvement anti-TNF treatment offers better outcome compared to cDMARDS. Other treatments such as Janus kinus inhibitors, IL-17, PDE-4 inhibitors and bisphosphonates have been used. There are no agreed composite measures to assess disease activity in SAPHO and repeat imaging may be used to guide treatment.

**Key learning points:**

One needs to keep a high index of suspicion of SAPHO in patients with isolated sternoclavicular joint disease especially if there is co-existant skin disease. Most commonly affected skeletal site is the anterior chest wall, including sternoclavicular, manubrosternal and costosternal joints. Axial skeleton, including the spine and sacroiliac joints are other common sites.

Palmoplantar psoriasis and severe acne are the main skin manifestations of SAPHO and should act as an alert

The time interval between the occurrence of initial skin and arthritis/osteitis symptoms in SAPHO can be many years. In the majority of cases this is usually within two years.

Exacerbation of osteoarticular and dermatologic manifestations are often unrelated to each.

Magnetic resonance imaging (MRI) is more sensitive in picking up asymptomatic and early lesions.

